# Diaper need in the United States: A nationally representative study during the COVID-19 pandemic

**DOI:** 10.1016/j.heliyon.2024.e31344

**Published:** 2024-05-16

**Authors:** Emily H. Belarmino, Carollyne M. Conway, Jane Kolodinsky, Kaya M. Daylor, Emma Spence

**Affiliations:** a Department of Nutrition and Food Sciences, University of Vermont, 350 Carrigan Wing, Marsh Life Science, 109 Carrigan Drive, Burlington, VT, 05405, USA; b Gund Institute for Environment, University of Vermont, Farrell Hall, 210 Colchester Avenue, Burlington, VT, 05405, USA; c Environmental Science Program, College of Agriculture and Life Sciences, Morrill Hall, University of Vermont, Burlington, VT, 05405, USA; d Department of Community Development and Applied Economics, University of Vermont, 202 Morrill Hall, Burlington, VT, 05405, USA; e Center for Rural Studies, University of Vermont, 206 Morrill Hall, Burlington, VT, 05405, USA; f Data Science Program, Department of Mathematics and Statistics, E220 Innovation Hall, 82 University Place, University of Vermont, Burlington, VT, 05405, USA; g Food Systems Program, University of Vermont, 236 Marsh Life Science, 109 Carrigan Drive, Burlington, VT, 05405, USA

## Abstract

**Background:**

Diapers represent a unique financial burden for those with young children. Pre-pandemic, approximately one in three U.S. households with young children reported diaper need or an insufficient supply of diapers. To support this population, policymakers, clinicians, and service providers need a better understanding of the groups most commonly affected and the ways that families cope with deprivation.

**Methods:**

An online survey was administered between February and July 2021 to a national sample of U.S. caregivers of at least one child aged 0–4 years in diapers (n = 881), investigating diaper need, diaper access, and how household expenses are balanced and prioritized vis-à-vis diapers. We use bivariate and multivariable models to assess factors associated with diaper need, and examine coping strategies, tradeoffs made to purchase diapers, and how caregivers would reallocate their money if diapers were accounted for.

**Findings:**

The prevalence of diaper need (46 %) exceeded pre-pandemic estimates. Diaper need was more prevalent among Hispanic respondents, lower income respondents, cloth diaper users, those with more than one child in diapers, caretakers with depression, and those with a negative financial change in the past year. Caretakers with diaper need were more likely to utilize a range of resources to access diapers and to use diapers for longer than desired to extend their supply. Those with diaper need also made more economic tradeoffs to afford diapers and indicated that they would reallocate resources to cover other unmet basic needs if they did not have to buy diapers.

**Interpretation:**

Our results indicate elevated levels of diaper need even after the U.S. economy had largely rebounded and raise concerns that inequities in diaper access may impact families’ abilities to meet other basic needs. Diapers may be an important target for policies and interventions aimed at improving the well-being of families with young children.

## 1 Introduction

Diaper purchases are essential for families with young children, and represent a financial burden not faced by most other types of households. More households with young children also live in poverty; in 2019, 15.4 % of children under age six lived in households earning less than the Federal poverty line (FPL) [1] compared to 10.5 % of all Americans [2]. A monthly supply of diapers for one child is estimated to cost $70–80 [3] or over 3 % of the monthly income for a family of four earning at the poverty line [4].

A growing body of literature links diaper need – the lack of an adequate supply of diapers to keep your child clean, dry, and healthy [3] – with adverse health outcomes, including increased pediatric visits for diaper dermatitis and urinary tract infections [5], poor child sleep [6], and poor maternal mental health [[7], [8], [9], [10]]. Diaper need can also interfere with parents' abilities to work and children's access to education [[11], [12], [13]], as most childcare centers require caregivers to provide diapers. In several studies conducted prior to the COVID-19 pandemic, about one in three mothers of a young child reported diaper need [5,7,11,14].

Although diapers are a basic hygiene item, the priority given to diapering may depend on, or compete with, other basic needs including food, housing, healthcare, and personal safety, especially among low-income households. For example, qualitative research by Randles [15] found that low-income mothers diligently and creatively managed limited resources to provide diapers, most commonly sacrificing their own needs. This has been corroborated by quantitative studies documenting reliance on diverse sources of diapers among low-income households [9,11,14]. Research with low-income recipients of diapers from one community-based diaper bank found associations between high levels of diaper need and unmet needs for housing, utilities, medical expenses, transportation, food, and other nonfood household items [12]. So far, few peer-reviewed studies have investigated how those with young children prioritize diapers vis-à-vis other needs.

Since 2020, households with young children have been disproportionately impacted by the COVID-19 pandemic. According to a nationally representative survey conducted in September 2020, four in 10 parents (40.3 %) living with a child under age six reported that their family experienced loss of work or work-related income during the first six months of the pandemic. To cope with these losses and make ends meet, parents reported spending less on food (34.4 %); using all or most of their savings (26 %); increasing credit card debt (25.5 %); taking money out of retirement, college, or other long-term savings (14.4 %); borrowing money from family or friends (12.7 %); and pawning or selling possessions (11.3 %) [16]. Analysis of the Census Bureau's Household Pulse Survey found adults in households with children were more likely to have trouble paying usual household expenses than those without children [17].

Only one peer-reviewed population-level study has estimated diaper need since the onset of the COVID-19 pandemic [9]. This study found that 36 % of Massachusetts households with young children in diapers reported diaper need. Given that Massachusetts spends more on health care and social services than the national average [18], it is possible that baseline diaper need was lower in the state or that the safety net response in Massachusetts was stronger than other states.

This study had two primary objectives. First, we aimed to document the level of diaper need experienced during the COVID-19 pandemic in a national sample and identify characteristics of those at increased risk of diaper need. We anticipated that levels of diaper need would be higher than previously reported, and that pandemic-related hardship would be associated with elevated rates of diaper need. Second, we aimed to describe and evaluate how caregivers cope with and balance the need for diapers with other household needs. To do this, we examined coping strategies, tradeoffs made to purchase diapers, and how caregivers would prioritize their money if diapers were already accounted for. Drawing from the limited research on the experience of living with diaper need [9,11,12,14,15,19], we expected that caregivers with diaper need would utilize more demanding strategies to access diapers, report sacrificing other basic needs to purchase diapers, and prioritize money saved on diapers for essential bills and products.

## 2 Materials and methods

Data are from a cross-sectional, web-based survey conducted between February 25, 2021 and July 4, 2021. The survey was designed to measure life challenges experienced by families with young children across the United States, including diaper need and challenges with other basic needs (Supplementary File 1).

### 2.1 Population

Qualtrics, a survey research firm, fielded the survey to a national research panel of adult caregivers (age 18+ years) with at least one child aged 0–4 years. Qualtrics partners with online sample providers to supply respondents that meet pre-specified criteria. For this project, target recruitment quotas were set for race, ethnicity, and income. Race and ethnicity quotas were proportional to the U.S. population based on the American Community Survey (ACS) 5-year 2019 data [20]. The income quota conservatively aimed for 45 % of the sample below 200 % of the FPL but this was not achieved due to issues with survey distribution; approximately 60 % of the sample fell below this threshold.

### 2.2 Measures

#### 2.2.1 Demographics and personal factors

The demographic factors collected included age, gender, race, ethnicity, household income in 2020, level of education, state of residence, rural or urban residence, whether the respondent is the sole adult caregiver in the household, and number of children in the household that wear diapers. Based on zip code, we used the Rural-Urban Commuting Area (RUCA) codes to classify respondents as living in a rural or urban area [21]. Respondents were asked to self-report their general health status using one of five categories: “excellent”, “very good”, “good”, “fair”, or “poor” [22]. To support investigation of experiences of diaper need among racial and ethnic minoritized groups, we recategorized respondents into four categories based on their self-reported race and ethnicity: (1) Black or African American (includes individuals of Hispanic and non-Hispanic origin); (2) Hispanic (excludes those who also identified as Black or African American), (3) non-Hispanic White, and (4) Other minoritized racial group. Individuals included in the final group self-identified as members of the following racial groups: American Indian or Alaskan Native, Asian, Native Hawaiian or other Pacific Islander, other race, and multiple races.

#### 2.2.2 Pandemic-related hardship

To assess pandemic-related changes in financial situation, respondents were asked whether their financial situation was “better off”, “worse off”, or “about the same” as it was at the same time the previous year. Depressive symptomatology was measured using the validated, 20-item Center for Epidemiologic Studies Depression (CES-D) scale [23,24]. We employed standardized scoring, with a score of ≥16 points used to classify participants as depressed. Given the unique childcare needs of households with young children, respondents were asked “If you work or would like to work outside of the home, do you have a childcare arrangement that supports your child's development and meets your needs?” Respondents who answered “No” were determined to have unmet needs for childcare and those who responded “Yes” were classified as having their childcare needs met.

#### 2.2.3 Diaper need

Only respondents who expressed that they had one or more children in diapers were included in the analysis. This was determined using the question “How many children in your household wear diapers (including pull ups) during the day or at night?” Among the people who had children in diapers, diaper need was assessed based on the survey question “Do you ever feel that you do not have enough diapers to change them as often as you would like?” Participants who responded “Yes” were labeled as having diaper need, whereas those who responded “No” were determined to have no diaper need [7]. Respondents were also asked what type of diapers they have used and could select disposable and/or cloth.

#### 2.2.4 Diaper sources and coping strategies

The survey asked respondents to report all of their sources of diapers from a list of five options: the store, an agency or support organization, friends or family, the doctor's office, or other. When possible, “other” responses were recoded into the other categories. To evaluate coping strategies, the survey asked, “Do you ever do any of these things so that your supply of diapers lasts longer? Choose all that apply.” Response options included stretching the diapers that you have (i.e., using fewer diapers to extend the supply), putting your child in underwear before they are ready, letting your child go without diapers or underwear, using cloth diapers, or none of the above.

#### 2.2.5 Tradeoffs and priorities

Tradeoffs were determined using the question “Which of the following have you done to ensure you could afford enough diapers? Choose all that apply.” Participants were provided with a list of 21 response options. Priorities for goods and services – outside of diapers – that a household deems important were determined by asking “If you were to receive diapers at no cost, which household expenses would you spend the extra money on? Please rank the top five things from the list below. Rank the first item 1, the second item 2, the third item 3, the fourth item 4, and the fifth item 5.” Respondents were limited to ranking five of 21 response options. Ranked items were categorized as needs that would be prioritized.

#### 2.2.6 Statistical methods

The survey was completed by 1125 individuals. We restricted the number for these analyses to respondents with at least one child in diapers who answered the question on diaper need (*n* = 881). Descriptive statistics were generated for demographic factors.

Bivariate analyses (chi-square tests with pairwise z-tests) were used to compare sample characteristics for respondents with and without diaper need. Because recruitment targets for the income quota were not achieved, we also examined bivariate relationships with the data weighted to reflect the national income distribution for households with at least one child aged 0–4 years [20]. Weighting the data did not substantially impact results thus the survey weight was not applied for any analyses. A multivariable logistic regression model, including variables from bivariate analyses that were significant at *p* < 0.05, was used to examine the association between diaper need and socio-demographic characteristics. Following Belarmino et al. [9], we used logistic regression, adjusting for the same covariates in our initial model, to assess relationships between diaper need and three types of hardship during the pandemic: a change in financial situation in the past year, screening positive for depression, and not having adequate childcare.

We also used chi-square tests to compare caregivers with and without diaper need with respect to (1) strategies used to access diapers, (2) tradeoffs made to purchase diapers, and (3) how financial resources would be prioritized if diapers were already accounted for. Data were analyzed and visualized using SPSS Statistics version 28.0 and R. We set significance for all tests at *p* < 0.05.

## 3 Results

Descriptive statistics for survey respondents (n = 881) are shown in Table 1. About half of respondents were 35 years or older (56.2 %), identified as female (53.7 %), and were non-Hispanic White (56.0 %). Most shared the home with at least one other adult (89.7 %), and lived in an area classified as urban (88.0 %). About one in four respondents (26.9 %) had more than one child in diapers. Most (77.4 %) had only used disposable diapers. Like the U.S. population [25], the largest proportion of respondents lived in the South Census Region (43.9 %). With respect to hardship experienced during the pandemic, 66.0 % screened positive for depression, 32.8 % reported a lack of adequate childcare, and 66.1 % reported being worse off or about the same financially as they were at the same time in the preceding year.

**Table 1:** Characteristics of U.S. caregivers of children aged 0–4 years in diapers (n = 881).

Characteristic	n	%
Respondent characteristics		
Age		
18–24 years	74	8.5
25–34 years	309	35.4
35–44 years	406	46.5
≥45 years	85	9.7
Gender		
Female	464	53.7
Not female	400	46.3
Race and Ethnicity^a^		
Black or African American	192	21.8
Hispanic	152	17.3
Non-Hispanic White	493	56.0
Other minoritized racial group	44	5.0
Education level		
Some high school or high school degree	172	19.9
Some college, associate degree, technical school, or apprenticeship	254	29.4
Bachelor's degree	226	26.2
Postgraduate or professional degree	211	24.4
Household income in 2020		
≤ $24,999	193	22.0
$25,000-$49,999	226	25.7
$50,000-$99,999	265	30.2
≥ $100,000	194	22.1
Area type		
Urban	764	12.0
Rural	104	88.0
Census Region		
Midwest	133	15.1
Northeast	183	20.8
South	387	43.9
West	178	20.2
Single-adult household		
Yes	90	10.3
No	783	89.7
Number of children that wear diapers		
1 child	644	73.1
≥2 children	237	26.9
Types of diapers used		
Disposable only	584	77.4
Cloth only	34	4.5
Both disposable and cloth	137	18.1
Overall health status		
Excellent	178	20.4
Very good	304	34.8
Good	238	27.2
Fair	117	13.4
Poor	37	4.2
Hardship during the pandemic		
Financial situation compared to one year ago		
Better off	297	33.8
About the same	313	35.6
Worse off	268	30.5
Screened positive for depression (CES-D)		
Yes	524	66.0
No	270	34.0
Inadequate childcare situation		
Yes	255	32.8
No	522	67.2

Note. Due to missing data sample sizes differed for the following variables: Age (n = 874), Gender (n = 864), Education level (n = 863); Household income in 2020 (n = 878); Area type (n = 868); Single-adult household (n = 873), Types of diapers used (n = 755), Overall health status (n = 874), Financial situation compared to one year ago (n = 878), Screened positive for depression (n = 794), and Inadequate childcare arrangement (n = 777).

a The “Other minoritized racial group” category is comprised of individuals from the following groups: American Indian or Alaskan Native alone (n = 4), Asian alone (n = 10), Native Hawaiian or Other Pacific Islander alone (n = 2), Other race alone (n = 9), Multiple races (n = 19).

### 3.1 Characteristics associated with diaper need

Overall, 46.1 % of respondents reported diaper need (Table 2). When data were weighted to reflect the national income distribution of the target population, 44.0 % of respondents reported diaper need (data not shown). Among demographic associations assessed in bivariate analyses, race and ethnicity (p < 0.001), income (p < 0.001), number of children in diapers (p < 0.001), and types of diapers used (p < 0.001) were all associated with diaper need. Specifically, Hispanic respondents, those with a household income < $100,000 in 2020, those with two or more children in diapers, and those who sometimes or always used cloth diapers had higher prevalence of diaper need compared to respondents who were non-Hispanic White, had a household income ≥ $100,000, had only one child in diapers, and used only disposable diapers. Diaper need was not associated with caregiver age, gender, education level, rural compared to non-rural residence, Census region, whether the respondent was the only adult in the household, or the overall health status of the caregiver.

**Table 2:** Bivariate associations between diaper need and caregiver characteristics^a^ (n = 881).

Characteristic	% with diaper need	% without diaper need	*P* value
All	46.1	53.9	
Respondent characteristics			
Age			0.151
18–24 years	54.1	45.9	
25–34 years	48.9	51.1	
35–44 years	42.4	57.6	
≥45 years	43.5	56.5	
Gender			0.553
Female	46.8	53.2	
Not female	44.8	55.3	
Race and Ethnicity^a^			<0.001
Black or African American	49.5	50.5	
Hispanic^b^	59.2	40.8	
Non-Hispanic White^b^	40.2	59.8	
Other minoritized racial group	52.3	47.7	
Education level			0.090
Some high school or high school degree	50.6	49.4	
Some college, associate degree, technical school, or apprenticeship	46.5	53.5	
Bachelor's degree	47.3	52.7	
Postgraduate or professional degree	38.4	61.6	
Household income in 2020			<0.001
≤ $24,999 ^b^	53.4	46.6	
$25,000-$49,999^c^	54.4	45.6	
$50,000-$99,999 ^d^	45.7	54.3	
≥ $100,000 ^b,c,d^	30.4	69.6	
Area type			0.276
Urban	45.3	54.7	
Rural	51.0	49.0	
Census Region			0.765
Midwest	48.1	51.9	
Northeast	46.4	53.6	
South	44.2	55.8	
West	48.3	51.7	
Single-adult household			0.137
Yes	53.3	46.7	
No	45.1	54.9	
Number of children that wear diapers			<0.001
1 child^b^	42.5	57.5	
≥2 children^b^	55.7	44.3	
Types of diapers used			<0.001
Disposable only^b,c^	40.4	59.6	
Cloth only^b^	70.6	29.4	
Both disposable and cloth^c^	63.5	36.5	
Overall health status			0.096
Excellent	48.3	51.7	
Very good	43.1	56.9	
Good	42.9	57.1	
Fair	56.4	43.6	
Poor	51.4	48.6	
Hardship during the pandemic			
Financial situation compared to one year ago			<0.001
Better off^b^	42.4	57.6	
About the same^c^	35.5	64.5	
Worse off^b,c^	62.7	37.3	
Screened positive for depression (CES-D)			<0.001
Yes^b^	59.9	40.1	
No^b^	19.6	80.4	
Inadequate childcare situation			0.130
Yes	51.0	49.0	
No	45.2	54.8	

Note. Comparisons are based on chi-square analyses. Due to missing data sample sizes differed for the following analyses: Age (n = 874), Gender (n = 864), Education level (n = 863); Household income in 2020 (n = 878); Area type (n = 868); Single-adult household (n = 873), Types of diapers used (n = 755), Overall health status (n = 874), Financial situation compared to one year ago (n = 878), Screened positive for depression (n = 794), and Inadequate childcare arrangement (n = 777).

^b, c, d^ Categories are significantly different from each other at p < 0.05.

a The “Other minoritized racial group” category is comprised of individuals from the following groups: American Indian or Alaskan Native alone (n = 4), Asian alone (n = 10), Native Hawaiian or Other Pacific Islander alone (n = 2), Other race alone (n = 9), Multiple races (n = 19).

Two of the three forms of pandemic-related hardship were associated with diaper need. A respondent's financial situation at the time of the survey compared to one year previously was significantly associated with diaper need (p < 0.001), with those who reported being worse off having the highest prevalence of diaper need (62.7 %), followed by those who reported being better off (42.4 %). Those whose financial situation was “about the same” had the lowest prevalence of diaper need (35.5 %). Respondents who screened positive for depression had a higher prevalence of diaper need than those that did not (p < 0.001). A respondent's childcare situation was not associated with diaper need.

Results of multivariable logistic regression models are presented in Table 3. The model examining the relationship between personal factors and diaper need (Model 1) found Hispanic respondents, those with more than one child in diapers, those who used cloth diapers sometimes or always, and those with a household income < $100,000 to be more likely to report diaper need than non-Hispanic White respondents (odds ratio [OR] 1.691, confidence interval [95 % CI] 1.116–2.562, p = 0.013), those with only one child in diapers (OR 1.663, 95 % CI 1.177–2.350, p = 0.004), those who used disposable diapers only (for cloth only OR 3.585, 95 % CI 1.638–7.846, p = 0.001 and for both disposable and cloth OR 2.308 95 % CI 1.546–3.446, p < 0.001), and those with an annual household income ≥ $100,000 (for ≤ $24,999 OR 2.593, 95 % CI 1.600–4.202, p < 0.001; for $25,000 - $49,999 OR 2.570, 95 % CI 1.613–4.093, p < 0.001; and for $50,000 - $99,999 OR 1.659, 95 % CI 1.053–2.614, p = 0.029).

**Table 3:** Multivariable logistic regression examining personal factors and pandemic-related hardship associated with diaper need during the COVID-19 pandemic among respondents with at least one child aged 0–4 years in diapers living in their household.^a^

Variables	Model 1: Personal characteristics		Model 2: Financial change in the preceding year		Model 3: Depression during the pandemic		Model 4: Inadequate childcare during the pandemic		Model 5: All three forms of hardship during the pandemic	
	OR (95 % CI)	*P* value	OR (95 % CI)	*P* value	OR (95 % CI)	*P* value	OR (95 % CI)	*P* value	OR (95 % CI)	*P* value
Respondent characteristics										
Race and Ethnicity										
Black or African American	1.325 (0.899–1.952)	0.154	1.310 (0.883–1.944)	0.180	1.444 (0.939–2.219)	0.094	1.468 (0.970–2.221)	0.069	1.504 (0.949–2.384)	0.082
Hispanic	**1.691 (1.116–2.562)**	**0.013**	**1.601 (1.045–2.453)**	**0.031**	**1.686 (1.069–2.661)**	**0.025**	**1.697 (1.092–2.637)**	**0.019**	1.634 (0.995–2.681)	0.052
Non-Hispanic White	Referent		Referent		Referent		Referent		Referent	
Other minoritized racial group	1.273 (0.633–2.563)	0.498	1.289 (0.626–2.657)	0.491	1.585 (0.733–3.427	0.242	1.559 (0.713–3.407)	0.266	1.877 (0.776–4.537)	0.162
≥2 children in diapers	**1.663 (1.177–2.350)**	**0.004**	**1.684 (1.181–2.400)**	**0.004**	1.364 (0.940–1.982)	0.103	**1.951 (1.333–2.854)**	**<0.001**	**1.563 (1.026–2.382)**	**0.037**
Types of diapers used										
Disposable only	Referent		Referent		Referent		Referent		Referent	
Cloth only	**3.585 (1.638–7.846)**	**0.001**	**3.572 (1.611–7.918)**	**0.002**	2.175 (0.973–4.861)	0.058	**3.598 (1.585–8.166)**	**0.002**	2.258 (0.959–5.320)	0.062
Both disposable and cloth	**2.308 (1.546–3.446)**	**<0.001**	**2.249 (1.496–3.382)**	**<0.001**	**2.349 (1.510–3.655)**	**<0.001**	**2.149 (1.408–3.282)**	**<0.001**	**2.082 (1.296–3.343)**	**0.002**
2020 household income										
≤ $24,999	**2.593 (1.600–4.202)**	**<0.001**	**2.224 (1.346–3.677)**	**0.002**	1.645 (0.952–2.840)	0.074	**2.482 (1.465–4.205)**	**<0.001**	**1.582 (0.861–2.907)**	**0.139**
$25,000 - $49,999	**2.570 (1.613–4.093)**	**<0.001**	**2.224 (1.369–3.611)**	**0.001**	**1.732 (1.026–2.925)**	**0.040**	**2.395 (1.457–3.935)**	**<0.001**	1.670 (0.942–2.963)	0.079
$50,000 - $99,999	**1.659 (1.053–2.614)**	**0.029**	1.569 (0.989–2.491)	0.056	1.442 (0.868–2.394)	0.158	1.609 (1.001–2.588)	0.050	1.389 (0.815–2.368)	0.227
$100,000 or more	Referent		Referent		Referent		Referent		Referent	
Hardship during the pandemic										
Financial situation compared to one year ago										
Better off			1.236 (0.842–1.815)	0.279					1.468 (0.925–2.329)	0.104
About the same			Referent						Referent	
Worse off			**2.526 (1.727–3.693)**	**<0.001**					**2.251 (1.435–3.532)**	**<0.001**
Screened positive for depression					**4.575 (3.091–6.772)**	**<0.001**			**4.069 (2.656–6.233)**	**<0.001**
Inadequate childcare situation							1.036 (0.727–1.475)	0.846	0.982 (0.66–1.459)	0.930

a Due to missing data sample sizes differed for each model: Model 1: n = 752; Model 2: n = 749; Model 3: n = 684; Model 4: n = 660; Model 5: n = 599.

When the model was expanded to incorporate changes to the respondent's financial status over the preceding 12 months (Model 2), screening positive for depression (Model 3), not having adequate childcare during the pandemic (Model 4), or all three forms of hardship during the pandemic (Model 5). Compared to Model 1, fewer personal factors were related to diaper need in the four subsequent models. In Model 2, respondents with a household income between $50,000 and $99,999 did not have greater diaper need than those with an annual household income ≥ $100,000. In Model 3, the number of children in diapers was not associated with diaper need, those who used cloth diapers only did not have more diaper need than those who exclusively used disposable diapers, and those with incomes ≤ $24,999 and between $50,000 and $99,999 did not have greater diaper need than those with an income ≥ $100,000. In Model 4, those with an income between $50,000 and $99,999 did not have greater diaper need than those with an income ≥ $100,000. Finally, in Model 5, Hispanic respondents did not have greater diaper need than non-Hispanic White respondents, exclusive cloth diaper users did not have greater diaper need than exclusive disposable diaper users, and those with an income between $20,000 and $99,999 did not have more diaper need than those with an income ≥ $100,000.

The worsening of a respondent's financial status over the preceding 12 months was associated with diaper need when considered in a separate model (Model 2; OR 2.526, 95 % CI 1.727–3.693, p < 0.001) and when considered together with other forms of hardship during the pandemic (Model 5; OR 2.251, 95 % CI 1.435–3.532, p < 0.001). Similarly, screening positive for depression was associated with diaper need when considered in a separate model (Model 3; OR 4.575, 95 % CI 3.091–6.772, p < 0.001) and when considered together with other forms of hardship (Model 5; OR 4.069, 95 % CI 2.656–6.233, p < 0.001). Not having adequate childcare during the pandemic was not significantly associated with diaper need in any model.

### 3.2 How caregivers cope and balance the need for diapers with other needs

**Fig. 1:**
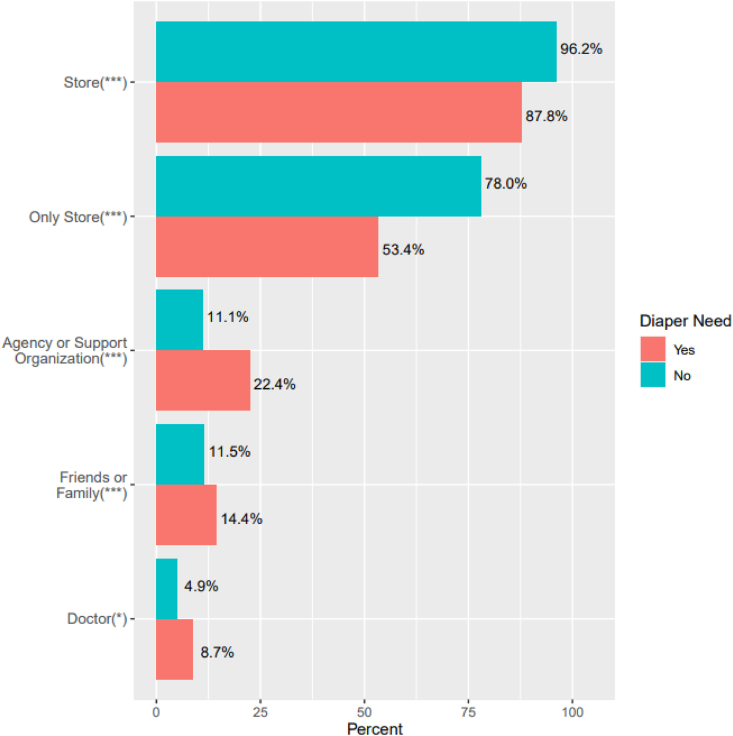
Sources of diapers used by caregivers with and without diaper need (n = 869) *p < 0.05; ***p < 0.001.

Overall, the store was the most common source of diapers (92.3 %) followed by friends and family (20.6 %), and an agency or support organization (16.3 %), data not shown. Sources of diapers by diaper need status are presented in Fig. 1. Compared to respondents with diaper need, those without diaper need were significantly more likely to acquire all their diapers from the store (78.0 % vs. 53.4 %, p < 0.001) and were significantly less likely to use each of the social and community resources to acquire diapers (p < 0.05).

**Fig. 2:**
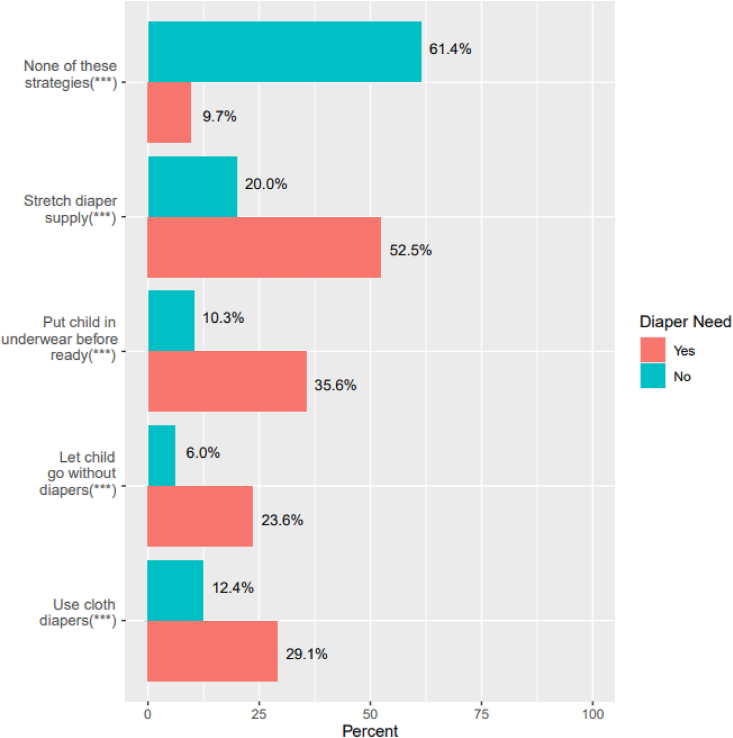
Coping strategies used by caregivers with and without diaper need to extend their supply of diapers (n = 868) ***p < 0.001.

Coping strategies to extend one's diaper supply are presented in Fig. 2. The most used strategy was stretching the diapers that one has (35.0 %), followed by putting a child in underwear before they are ready (22.0 %), using cloth diapers (20.2 %), and letting a child go without diaper or underwear (14.2 %), data not shown. Most respondents with diaper need reported using one or more of these strategies (90.3 %) compared to 61.4 % of respondents without diaper need who reported none of these strategies (p < 0.001).

Most respondents reported cutting back on at least one expense to afford diapers (62.3 %), with those with diaper need significantly more likely to report doing so (88.4 % vs. 40.3 %, p < 0.001; data not shown). Table 4 presents tradeoffs that caregivers with and without diaper need reported making to afford diapers. The most common tradeoffs reported were reducing use of utilities (26.9 %); purchasing lower cost foods (27.8 %); cutting back on clothing purchases (27.1 %); cutting back on home or car repairs (24.0 %); cutting back on media for the home (21.0 %); and cutting back on the amount of food purchased (20.7 %). Those with diaper need were more likely to report making each of the 20 tradeoffs queried.

**Table 4:** Tradeoffs that caregivers with and without diaper need reported to be able to afford diapers (n = 866).

Tradeoff	% all respondents	% with diaper need	% without diaper need	*P* value
Purchased lower cost foods	27.8	37.5	19.7	<0.001
Cut back on clothing purchases	27.1	37.0	18.9	<0.001
Reduced use of utilities (electric, gas, water, telephone, internet, etc.)	26.9	43.5	13.0	<0.001
Cut back on home or car repairs	24.0	39.2	11.3	<0.001
Cut back on media (cable, streaming service, etc.) for your home	21.0	31.9	11.9	<0.001
Cut back on the amount of food you buy	20.7	28.1	14.4	<0.001
Cut back on other transportation expenses, including gas or bus fares	18.0	27.6	10.0	<0.001
Did not pay the full amount of a bill, rent, or mortgage payment	17.1	27.8	8.1	<0.001
Cut back on other entertainment for you or other adults in your household	16.4	19.7	13.6	0.015
Cut back on purchases of household cleaning supplies	15.7	23.3	9.3	<0.001
Cut back on purchases of personal hygiene products (soap, toilet paper, period supplies, or oral healthcare products like toothpaste or toothbrushes)	12.7	20.0	6.8	<0.001
Did not go to the doctor or hospital when you or a member of your household were ill or injured	11.9	20.3	4.9	<0.001
Cancelled or reduced use of childcare/preschool	11.7	17.7	6.6	<0.001
Missed a preventive health, well child, or dental care visit	9.4	14.7	4.9	<0.001
Cut back on purchases of education or play items for your children, such as books or toys	9.4	12.9	6.4	<0.001
Cut back on purchases of diaper rash creams, powders, wipes, and other diaper care products	9.1	15.4	3.8	<0.001
Cut back on prescription medication for you or a member of your household	8.7	14.9	3.4	<0.001
Cut back on health insurance coverage	7.6	13.4	2.8	<0.001
Cut back on education for you or other adults in your household	6.2	9.4	3.6	<0.001
Cut back on purchases of over-the-counter medications or first aid supplies	6.1	11.1	1.9	<0.001

**Fig. 3:**
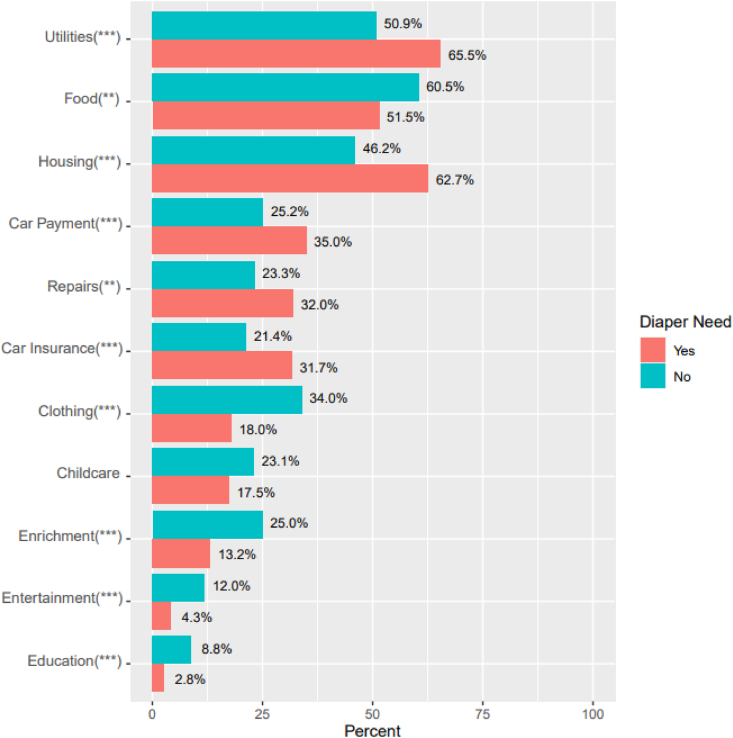
Needs that would be prioritized if the cost of diapers were accounted for, by diaper need status (n = 862) **p < 0.01; ***p < 0.001.

Respondents were asked the top five things that they would spend the extra money on if they were to receive diapers at no cost (Fig. 3). Over half indicated that they would prioritize the money for food (56.4 %), utilities (57.5 %), and/or housing (53.7 %). Respondents with diaper need were more likely to report that they would prioritize the money for utilities (65.5 % compared to 50.9 %, p < 0.001), housing (62.7 % compared to 46.2 %, p < 0.001), car payments (35.0 % compared to 25.2 %, p < 0.001), home or car repairs (32.0 % compared to 23.3 %, p = 0.005), and car insurance (31.7 % compared to 21.4 %, p < 0.001). Respondents without diaper need were more likely to report that they would prioritize the money for food (60.5 % compared to 51.5 %, p = 0.01), clothing (34.0 % compared to 18.0 %, p < 0.001), educational or play items for children (25.0 % compared to 13.2 %, p < 0.001), entertainment for themselves or other adults in the household (12.0 % compared to 4.3 %, p < 0.001), and education for themselves or other adults in the household (8.8 % compared to 2.8 %, p < 0.001).

## 4 Discussion

To our knowledge, this was the first peer-reviewed national study to examine diaper need during the COVID-19 pandemic, with results potentially important for diaper need relief policy discussions and community health. Notably, 46.1 % of respondents indicated diaper need, which exceeds pre-pandemic estimates, but is consistent with the findings of a survey conducted by the National Diaper Bank Network in 2023 [13] and reports of increased demand for diapers from diaper banks [26]. This also aligns with evidence of families with children experiencing increased hardship during the pandemic [17,[27], [28], [29]] even after unemployment rates had largely recovered from the peak in April 2020 [30]. Weighting our sample by income to match the U.S. population of households with children aged 0–4 years only slightly reduced the proportion of caregivers reporting diaper need (44.0 %); over two in five caregivers to a young child still struggled to access enough diapers.

Corroborating earlier studies, we found diaper need to be higher among Hispanic caregivers [9,11], and those who lived in low income households [9,11], had depressive symptoms [[7], [8], [9]], or experienced a worsening of their financial situation during the pandemic [9]. This study also found that caregivers with more than one child in diapers and those who sometimes or always used cloth diapers were more likely to report diaper need, likely reflecting the increased costs of diapering multiple children and the use of cloth diapers as a strategy to buffer hardship related to the costs of disposable diapers. After adjustment, associations between ethnicity, income, number of children in the household in diapers, use of cloth diapers, depressive symptoms, and a worsening financial situation during the pandemic mostly remained significant, although not all variables were related to diaper need in all models. This was the first peer-reviewed study on diaper need to include a similar number of caretakers who identify as female and male; most prior research has focused on mothers. Earlier studies found diaper need to be higher among caregivers in early adulthood [9,10]and who have limited education [9,11]. While we did not identify these relationships to be significant in the present analysis, the trends in the data parallel what has been documented previously.

Unlike Raver et al. [11], we did not identify a higher prevalence of diaper need among single parents. This is the second study conducted during the pandemic that did not find the number of adults in the household to be associated with diaper need [9]. Notably, only about 10 % of the samples for both studies reported that they were the only adult in their household. Nationally, an estimated 23 % of children lived in single-parent households before the pandemic [31]; however, emerging data suggest that the pandemic increased the number of shared households with implications for the household's economic stability and the overall wellness of household members [32]. Thus, it is unclear whether the study samples differed from the general population in this respect or if they accurately reflected the context during the pandemic.

Additionally, overall health status was not associated with diaper need. Prior research in Massachusetts found that having a household member (self or other) diagnosed with a chronic health condition was associated with diaper need [9]. Further, a study of under resourced caregivers with neurodiverse children conducted in early 2021 found 76 % to report some diaper need [10]. The present study did not ask about diagnoses among household members, but future studies should further explore links between diaper need and poor health and disability, particularly health conditions that may be more costly or time intensive to manage or treat. Although not a focus of this analysis, the very high prevalence of depression among this population is noteworthy, given the documented links between caregiver mental health and family well-being [33,34].

Another key contribution of this study is that it extends in-depth qualitative research and more limited quantitative research to evaluate how caregivers cope and balance the need for diapers with other household needs. As predicted, we observed that caregivers with diaper need utilized more logistically demanding strategies to diaper their children. Randles [15] refers to these laborious efforts to acquire and maintain a supply of diapers as a form of ‘inventive mothering’, or part of a set of innovative behaviors that poor mothers engage in to meet their children's most fundamental needs. Compared to respondents without diaper need, those with diaper need were more apt to utilize a range of social and community resources to access diapers, highlighting the importance of community-based safety nets for those experiencing this form of deprivation, especially in the absence of federal policy to alleviate diaper need. Yet, these resources are unlikely to be adequate given the limited number of families served by diaper banks [35] and the high levels of need.

We also found caregivers with diaper need to be more likely to engage in behaviors that would extend their supply of diapers. Over one-third of all caregivers and half with diaper need reported “stretching” the supply of diapers to make them last longer, a practice associated with diaper dermatitis and urinary tract infections. In light of previous research linking diaper need to child discomfort [10,12] and increased pediatric care visits for diaper dermatitis and urinary tract infections [5], the present findings suggest that an adequate supply of diapers may contribute to improved wellbeing and a reduced number of pediatric care visits. This research also supports the assertion by Shaffer et al. [10] that diaper need be considered a pediatric social determinant of health.

Previous research has documented that low-income households with young children have multiple challenges in managing basic needs [12] and there is some evidence that caregivers with diaper need cut back on other household essentials to afford diapers [11,13]. The findings of the present study add new dimensions to this literature and identify the other basic needs that caregivers compromise to buy diapers, with potential adverse health consequences. For example, to afford diapers, over one-third of respondents with diaper need reported cutting back on the quality of food purchased and over one-fourth reported reducing the quantity of food purchased. Diaper need has previously been associated with food insecurity [[8], [9], [10],^12^,^14^], but this is the first study to document caregivers reporting buying lower cost foods to afford diapers. Prior research has found food costs to be associated with nutrient density, with more nutritious diets costing more [36,37]. Those findings, together with the findings reported here, suggest that diaper need may constrain the purchase and consumption of nutritious foods. Extensive research has documented negative effects from food insecurity and poor diet quality on physical [[38], [39], [40]] and mental health [[41], [42], [43]].

Respondents’ predictions for how they would reallocate financial resources if they did not have to pay for diapers identified important differences between those with and without diaper need. Notably, respondents with diaper need were more likely to report that they would use the money saved to cover immediate living expenses (e.g., housing and utilities) and car costs (e.g., car payments and insurance). Reflecting prior fulfillment of basic physiological and safety needs, those without diaper need were more likely to report using the extra money on things that may add fun or enrichment to their lives, specifically clothing, entertainment, childcare, education, and food. The inclusion of food on this list may relate to buying more indulgent food and/or eating outside of the home more frequently rather than buying staples to achieve food security as two prior studies found recipients of free diapers through community diaper banks to report that receiving diapers allowed them to spend more money on food [12,44]. Our findings suggest that if households with diaper need received a diaper benefit, they would reallocate their household budget to meet other fundamental needs rather than to discretionary spending. This is especially timely as federal legislation to address diaper need has been proposed and is currently under discussion.

### 4.1 Limitations

The results of this research should be interpreted considering several limitations. The research was cross-sectional, limiting the ability to make statements about causal relationships between diaper need and associated characteristics and factors. We did not measure change in diaper need between pre-pandemic and during the pandemic due to the transient nature of diaper requirements. Unlike other basic needs required across life stages, diapers are typically only necessary during a child's early years. Thus, many households using diapers pre-pandemic no longer required them during the pandemic as their child gained skills in using a toilet and, likewise, households that added a baby or toddler during the pandemic experienced new or increased needs for diapers that they did not have pre-pandemic. Thus, cross-sectional research may be most appropriate for understanding this phenomenon. Second, the survey was only available in English and those without access to the internet or who are not literate in English would not have been able to take part. While 93 % of American adults use the internet, the use of an English-only online survey likely undercounted some groups at increased risk for diaper need (e.g., those with very low income, without high school degrees, and that do not speak English) [7,45]. Third, as with prior research on diaper need, this study used an experience-based measure of need. Research is needed to better understand how experience-based and objective measures of diaper need (e.g., reported number of diapers used by child age) align, as well as whether experience-based measures do indeed track the households with greatest diaper need. Fourth, our measure of household income was categorical. Thus, it was not possible to assess income as a percentage of the federal poverty guidelines, which account for household size. Fifth, our measure of financial disruption during the pandemic may not have captured caregivers who experienced a financial shock during the early months of the COVID-19 pandemic but then recovered in the subsequent months. We asked about changes to a household's financial situation since “this time last year”, but over 90 % of respondents completed the survey on or after March 11, 2021, the one year anniversary of when the World Health Organization declared COVID-19 a pandemic [46]. It is important to note, however, that any bias introduced by this measure would have biased results in the direction of undercounting, rather than overcounting hardship. Finally, all measures in this study are self-reported and may be subject to reporting bias. However, the fact that the survey was anonymous and fielded online may lessen this concern.

## 5 Conclusions

The results of this study suggest that diaper need increased during the COVID-19 pandemic and identify characteristics and forms of hardship associated with increased risk for diaper need. The findings also shed light on the complex and strategic decisions caregivers make about how to provide diapers for their children while managing other pressing household needs. For example, households with diaper need were more likely to report buying less food and lower quality food to afford diapers. Notably from public policy and clinical care perspectives, households with diaper need reported that they would reallocate their budget to alleviate other neglected basic needs if they were to receive diapers at no cost. This strongly suggests programs and policies designed to alleviate diaper need may have a multiplier effect for child and family health and wellbeing and that those who participate would use the support to enhance their basic security and dignity rather than purchase inconsequential items. Such evidence is critical to support investment in the social safety net, particularly considering long-standing negative perceptions and stigmatization that plague some safety net programs and their participants [47,48]. There is a need for further research on the impact of programs and policies to reduce diaper need on key economic and public health outcomes including healthcare expenditure, workforce participation, health behaviors like dietary intake, and child and caregiver health outcomes. Effective programs and legislation would be a boon for the millions of US families who make daily tradeoffs between purchasing diapers and meeting other basic needs.

## Data availability statement

The data associated with this study have not been deposited into a publicly available repository. The data will be made available upon request.

## Ethics declarations

Review and/or approval by an ethics committee was not needed for this study because the Institutional Review Board (IRB) Committee on Human Subjects Serving the University of Vermont and the University of Vermont Medical Center determined this study to be exempt from review and provided a waiver of documentation of consent. This waiver was granted as the research presented more than no more than minimal risk to participants and involved no procedures in which written consent is normally required outside the research context.

## CRediT authorship contribution statement

**Emily H. Belarmino:** Writing – review & editing, Writing – original draft, Supervision, Project administration, Methodology, Investigation, Funding acquisition, Formal analysis, Conceptualization. **Carollyne M. Conway:** Writing – review & editing, Investigation. **Jane Kolodinsky:** Writing – review & editing, Methodology, Conceptualization. **Kaya M. Daylor:** Writing – review & editing, Visualization, Formal analysis. **Emma Spence:** Writing – review & editing, Supervision, Investigation.

## Declaration of competing interest

The authors declare the following financial interests/personal relationships which may be considered as potential competing interests: 10.13039/100011633Emily H Belarmino reports financial support was provided by the University of Vermont’s Agricultural Experiment Station through the State of Vermont’s appropriation.. Emily H. Belarmino reports a relationship with US Department of Health and Human Services that includes: consulting or advisory. If there are other authors, they declare that they have no known competing financial interests or personal relationships that could have appeared to influence the work reported in this paper.
