# Supplementary material for: Diaper need in the United States: A nationally representative study during the COVID-19 pandemic
Source: Heliyon. 2024 May 16;10(10):e31344. doi: 10.1016/j.heliyon.2024.e31344 (PMC11130655; doi:10.1016/j.heliyon.2024.e31344)
Supplement: Multimedia component 1 [file mmc1.pdf]

Information Sheet and Consent

BASIC NEEDS AND FOOD SECURITY

Research Information Sheet

Title of Study: Food and nonfood needs among families with young children
Principal Investigator (PI): Emily Belarmino
Funder: University of Vermont College of Agriculture and Life Sciences

Introduction
You are being invited to take part in this research study because you are the primary caregiver of a child under 5 years old, have lived in the US since the start of 2020, and 18 years of age or older. This study is being conducted by Emily Belarmino at the University of Vermont.

Purpose
This study is being conducted to understand life challenges experienced by families with young children.

Study Procedures
If you take part in the study, you will be asked to take an online survey asking questions about your satisfaction with various aspects of your life, your experiences and priorities accessing food and other household items, and your health. The survey will take approximately 20 minutes. You are free to not answer any questions and remain in the study.

Benefits
As a participant in this research study, there may not be any direct benefit for you; however, information from this study may benefit other people now or in the future.

Risks
We will do our best to protect the information we collect from you and avoid any potential risk for an accidental breach of confidentiality.

Costs
There will be no costs to you for participation in this research study.

Compensation
For taking part in this research study and completing the entire survey you are compensated through your partnership with your survey company.

Confidentiality
All information collected about you during the course of this study will be stored with a code number. The code name will be kept separate from directly identifiable information, such as your zipcode, which is only available to the lead PI. We will keep the data for the duration of the study, approximately two years. The de-identified data may be shared with external collaborators and researchers for data analysis.

Voluntary Participation/Withdrawal
Taking part in this study is voluntary. You are free to not answer any questions or withdraw at any time. You may choose not to take part in this study, or if you decide to take part, you can change your mind later and withdraw from the study. However, your de-identified data cannot be withdrawn from the study and will be used.

Questions

If you have any questions about this study now or in the future, you may contact Emily Belarmino at 802-656-0540 or ehmorgan@uvm.edu (preferable). If you have questions or concerns about your rights as a research participant, then you may contact the Director of the Research Protections Office at (802) 656-5040.

It is recommended you print this information sheet for your records before continuing.

If you agree to participate in this research, please click “yes” below to begin taking the survey. Clicking “yes” on this form means that you have reviewed the information in this form, you have had a chance to ask questions, and you agree to join the study.

- Yes
- No

Screener

Have you lived in the United States since at least January 1st, 2020?

- Yes
- No

Are you the primary caregiver of a child under age 5 years who lives in your household?

- Yes
- No

Please select your age group:

- <18 years old
- 18-34 years old
- 35-54 years old
- 55 years and older

Are you of Hispanic, Latino, or Spanish origin?

- No, not of Hispanic, Latino, or Spanish origin
- Yes

What is your race?

Please choose **all** that apply:

- American Indian or Alaskan Native
- Asian
- Black or African American
- Native Hawaiian or Other Pacific Islander
- White
- Other race

What is your state?

How many family members, including yourself, reside in your household? We define family as everyone related to each other by blood, marriage or a marriage-like relationship including partners and foster children.

Screener - PVL

Was your total household income in 2020 before taxes less than ${e://Field/FPL}?

- Yes
- No

1.1 - BASIC NEEDS SATISFACTION

The purpose of this set of questions is to find out how you feel about various parts of your life. Please include the feelings you have now -- taking into account what has happened in the last year and what you expect in the near future.

How do you feel about...

	Unhappy	Mostly dissatisfied	Mixed (about equally satisfied and dissatisfied)	Mostly satisfied	Pleased
The physical comfort of your home -- heat, water, lighting, ventilation.	○	○	○	○	○
Your level of physical activity.	○	○	○	○	○
Your family life--your wife/husband, your marriage, your children, if any.	○	○	○	○	○
The chance you have to know people with whom you can really feel comfortable.	○	○	○	○	○
The extent to which you are developing yourself and broadening your life.	○	○	○	○	○
How secure you are from people who might steal or destroy your property.	○	○	○	○	○
The amount of respect you get from others.	○	○	○	○	○
Yourself.	○	○	○	○	○
The way you handle the problems that come up in your life.	○	○	○	○	○
How much you are accepted and included by others.	○	○	○	○	○
	Unhappy	Mostly dissatisfied	Mixed (about equally satisfied and dissatisfied)	Mostly satisfied	Pleased
The way other people [illegible]	○	○	○	○	○

The chance you have to enjoy pleasant or beautiful things.	○	○	○	○	○
The reliability of the people you depend on.	○	○	○	○	○
Your safety.	○	○	○	○	○
How creative you can be.	○	○	○	○	○
The amount of friendship and love in your life.	○	○	○	○	○
Your sex life.	○	○	○	○	○
Your own health and physical condition.	○	○	○	○	○
The amount of fun and enjoyment you have.	○	○	○	○	○
	Unhappy	Mostly dissatisfied	Mixed (about equally satisfied and dissatisfied)	Mostly satisfied	Pleased
The sleep you get.	○	○	○	○	○
How secure you are financially.	○	○	○	○	○
How dependable and responsible people around you are.	○	○	○	○	○
The extent to which your world seems consistent and understandable.	○	○	○	○	○
The extent to which your personal needs are met.	○	○	○	○	○
The way you spend your spare time, your non-working activities.	○	○	○	○	○

1.2 GENERAL MATERIAL HARDSHIP

We are also interested in some of the problems families face making ends meet. In the past 12 months, did you do or experience any of the following because there wasn't enough money? **Please select all that apply.**

- ☐ Did not pay the full amount of rent or mortgage
- ☐ Did not pay the full amount of a gas, oil, or electricity bill
- ☐ Telephone service was ever disconnected
- ☐ Gas or electricity was turned off
- ☐ Moved in with other people even for a little while because of financial problems
- ☐ Stayed in a shelter, in an abandoned building, an automobile or any other place not meant for regular housing, even for one night
- ☐ Was evicted from your home or apartment for not paying the rent or mortgage
- ☐ None of these

In the past 12 months, was there anyone in your household who needed to see a doctor or go to the hospital but couldn't because of the cost?

- ○ Yes
- ○ No

1.3 DIAPER NEED

How many children in your household wear diapers (including pull ups) during the day or at night?

- 0 children
- 1 child
- 2 children
- 3 children
- 4 or more children

Do you ever feel that you do not have enough diapers to change them as often as you would like?

- Yes
- No

Where do you get diapers? **Choose all that apply.**

- From the store
- From an agency or support organization
- From friends or family
- From the doctor's office
- ________ Other

Do you ever do any of these things so that your supply of diapers lasts longer? **Choose all that apply.**

- ☐ Stretch the diapers that you have
- ☐ Put your child in underwear before they are ready
- ☐ Let your child go without diapers or underwear
- ☐ Use cloth diapers
- ☐ None of the above

Have you ever cut back on your expenses to ensure you could afford enough diapers?

- ○ Yes
- ○ No

Which of the following have you done to ensure you could afford enough diapers? **Choose all that apply.**

- ☐ Did not pay the full amount of a bill, rent, or mortgage payment
- ☐ Reduced use of utilities (electric, gas, water, telephone, internet, etc.)
- ☐ Cut back on home or car repairs
- ☐ Cut back on other transportation expenses, including gas or bus fares
- ☐ Cut back on health insurance coverage
- ☐ Did not go to the doctor or hospital when you or a member of your household were ill or injured
- ☐ Missed a preventive health, well child, or dental care visit
- ☐ Cut back on prescription medication for you or a member of your household
- ☐ Cut back on purchases of over the counter medications or first aid supplies
- ☐ Cancelled or reduced use of childcare/preschool
- ☐ Cut back on the amount of food you buy
- ☐ Purchased lower cost foods
- ☐ Cut back on purchases of diaper rash creams, powders, wipes and other diaper care products
- ☐ Cut back on purchases of personal hygiene products (soap, toilet paper, period supplies, or oral healthcare products like toothpaste or toothbrushes)
- ☐ Cut back on purchases of household cleaning supplies
- ☐ Cut back on clothing purchases
- ☐ Cut back on purchases of educational or play items for your children, such as books or toys
- ☐ Cut back on education for you or other adults in your household
- ☐ Cut back on media (cable, streaming service, etc.) for your home
- ☐ Cut back on other entertainment for you or other adults in your household
- ☐ ________ Other:

Have you ever gone without diapers so that you could pay other expenses?

- Yes
- No

Which of the following expenses have you paid before buying diapers?
Choose all that apply.

- ☐ Housing (rent or mortgage payment)
- ☐ Utilities (electric, gas, telephone, etc.)
- ☐ Car payment
- ☐ Car insurance
- ☐ Home or car repairs
- ☐ Other transportation expenses, including gas or bus fares
- ☐ Health insurance
- ☐ Healthcare visits
- ☐ Prescription medication
- ☐ Over the counter medication or first aid supplies
- ☐ Child care/preschool
- ☐ Food
- ☐ Diaper rash creams, powders, wipes and other baby care products not including diapers
- ☐ Personal hygiene products (soap, toilet paper, period supplies, or oral healthcare products like toothpaste or toothbrushes)
- ☐ Household cleaning supplies
- ☐ Clothing
- ☐ Educational or play items for your child(ren) (books, toys)
- ☐ Education for you or other adults in your household
- ☐ Media (cable, streaming service, etc.)
- ☐ Entertainment for you or other adults in your household
- ☐ ________ Other:

If you work or would like to work outside of the home, do you have a childcare arrangement that supports your child’s development and meets your needs?

- Yes
- No
- Not applicable

Has/have your child(ren) ever missed childcare or preschool because you did not have a sufficient supply of diapers to send?

- Yes
- No
- Not applicable

If you were to receive diapers at no cost, which household expenses would you spend the extra money on? Please rank the top five things from the list below. Rank the first item 1, the second item 2, the third item 3, the fourth item 4, and the fifth item 5.

- [ ] Housing (rent or mortgage payment)
- [ ] Utilities (electric, gas, telephone, etc.)
- [ ] Car payment
- [ ] Car insurance
- [ ] Home or car repairs
- [ ] Other transportation expenses, including gas or bus fares
- [ ] Health insurance

☐ Healthcare visits

☐ Prescription medication

☐ Over the counter medication or first aid supplies

☐ Child care/preschool

☐ Food

☐ Diaper rash creams, powders, wipes and other baby care products not including diapers

☐ Personal hygiene products (soap, toilet paper, period supplies, or oral healthcare products like toothpaste or toothbrushes)

☐ Household cleaning supplies

☐ Clothing

☐ Educational or play items for your child(ren) (books, toys)

☐ Education for you or other adults in your household

☐ Media (cable, streaming service, etc.)

☐ Entertainment for you or other adults in your household

☐ Other: ______

What type of diapers have you used? **Choose all that apply.**

☐ Disposable
☐ Cloth

Do you have any additional comments or experiences related to diaper use

that you would like to share? Please use this space:

1.4 - HOUSEHOLD FOOD SECURITY

These next questions are about the food eaten in your household in the last 30 days and whether you were able to afford the food you need.

Now you are going to read several statements that people have made about their food situation. For these statements, report whether the statement was often true, sometimes true, or never true for your household in the last 30 days.

The first statement is, "The food that we bought just didn't last, and we didn't have money to get more." Was that often, sometimes, or never true for (you/your household) in the last 30 days?

- Often true
- Sometimes true
- Never true
- Don't know

“We couldn’t afford to eat balanced meals.” Was that often, sometimes, or never true for your household in the last 30 days?

- Often true
- Sometimes true
- Never true
- Don't know

In the last 30 days did you or other adults in your household ever cut the size of your meals or skip meals because there wasn't enough money for food?

- Yes
- No
- Don't know

In the last 30 days, how often did this happen?

- [ ] Days:
- Don't know

In the last 30 days, did you ever eat less than you felt you should because there wasn't enough money for food?

- Yes
- No
- Don't know

In the last 30 days, were you ever hungry but didn't eat because there wasn't enough money for food?

- Yes
- No
- Don't know

About how many cups of fruit (including 100% fruit juice) do you eat or drink each day?

Examples of 1 cup of fruit could be 1 small apple, 1 large banana, 1 large orange, 8 large strawberries, 1 medium pear, 2 large plums, 32 seedless grapes, 1 cup (8 oz.) of 100% juice, or 1/2 cup of dried fruit.

- None
- Less than 1/2 cup
- 1/2 cup
- 1 to 1 1/2 cups
- 2 to 2 1/2 cups
- 3 to 3 1/2 cups
- 4 or more cups

About how many cups of vegetables (including 100% vegetable juice) do you eat or drink each day?

Examples of 1 cup of vegetables could be 3 broccoli spears (5 in. long), 1 cup of cooked leafy greens, 2 cups of lettuce or raw greens, 12 baby carrots, 1 medium potato, 1 large sweet potato, 1 large ear of corn, 1 large raw tomato, or 2 large celery stalks.

- None
- Less than 1/2 cup
- 1/2 to 1 cup
- 1 1/2 to 2 cups
- 2 1/2 to 3 cups
- 3 1/2 cups to 4 cups
- 4 1/2 cups or more

SECTION 2 – ECONOMIC SITUATION

Which of the following best describes your household income range in 2020 before taxes? Household includes people currently living within your home, including family and non-family members.

- Less than $10,000 per year
- $10,000-$24,999 per year
- $25,000-$49,999 per year
- $50,000-$74,999 per year
- $75,000-$99,999 per year
- More than $100,000 per year

How many adults in your household have a job that earns wages?

Do you have a job that earns wages?

- Yes
- No

What is your hourly wage?

On average, how many hours per week do you work for wages?

Does any member of your household receive health insurance through an employer?

- Yes
- No

Does this health insurance cover all members of your household?

- Yes
- No

When it comes to your financial situation, would you say you are better off, worse off or about the same as you were this time last year?

- Better off
- Worse off
- About the same

In the past year, have you or any family member living with you, received assistance from the government for food, housing, utilities, education, or something else?

- Yes
- No

In the past year, have you or any family member living with you, received assistance from a non-profit organization for food, housing, utilities, education, or something else?

- Yes
- No

Has your household utilized any of the following programs in the last year? **Choose all that apply.**

- ☐ Food pantry or food shelf
- ☐ Food pickups of meals ready to eat (MRE)
- ☐ Housing assistance (subsidized housing, public housing, or the Housing Choice Voucher Program (Section 8))
- ☐ School Meal Program (in-school or dropoff/pickup)
- ☐ Supplemental Nutrition Assistance Program (SNAP or food stamps)
- ☐ Temporary Assistance for Needy Families (TANF)
- ☐ Special Supplemental Nutrition Program for Women, Infants, Children (WIC)
- ☐ Head Start
- ☐ Medicare
- ☐ Medicaid
- ☐ Children’s Health Insurance Program
- ☐ ______ Other:

Have you received any money from these sources in the last year? **Choose all that apply.**

- ☐ Federal stimulus check
- ☐ Friends or family
- ☐ Unemployment benefits
- ☐ None of the above

SECTION 3 - HEALTH OUTCOMES

Would you say your health in general is…

- Excellent
- Very good
- Good
- Fair
- Poor
- Don't know

Below is a list of the ways you might have felt or behaved. Please tell me how often you have felt this way during the past week.

	Rarely or none of the time (less than 1 day)	Some or a little of the time (1-2 days)	Occasionally or a moderate amount of the time (3-4 days)	Most or all of the time (5-7 days)
I was bothered by things that usually don’t bother me.	○	○	○	○
I did not feel like eating; my appetite was poor.	○	○	○	○
I felt that I could not shake off the blues even with help from my family or friends.	○	○	○	○
I felt I was just as good as other people.	○	○	○	○
I had trouble keeping my mind on what I was doing.	○	○	○	○
I felt depressed.	○	○	○	○
I felt that everything I did was an effort.	○	○	○	○
	Rarely or none of the time (less than 1 day)	Some or a little of the time (1-2 days)	Occasionally or a moderate amount of the time (3-4 days)	Most or all of the time (5-7 days)
I felt hopeful about the future.	○	○	○	○
I thought my life had been a failure.	○	○	○	○
I felt fearful.	○	○	○	○
My sleep was restless.	○	○	○	○
I was happy.	○	○	○	○
I talked less than				

	(less than 1 day)	time (1-2 days)	the time (3-4 days)	(5-7 days)
People were unfriendly.	○	○	○	○
I enjoyed life.	○	○	○	○
I had crying spells.	○	○	○	○
I felt sad.	○	○	○	○
I felt that people dislike me.	○	○	○	○
I could not get “going.”	○	○	○	○

How many cups of fruits and vegetables do you think the average adult should eat each day for good health?

Cups: [ ]

SECTION 4 - DEMOGRAPHIC QUESTIONS

How many adults age 18+ currently live in your household (including you)? Household includes people currently living within your home, including family and non-family members.

- 0
- 1
- 2
- 3
- 4
- 5 or more

How many children ages 5-17 currently live in your household?

- 0
- 1
- 2
- 3
- 4
- 5 or more

How many children under age 5 currently live in your household?

- 0
- 1
- 2
- 3
- 4
- 5 or more

What is the age of the youngest child in your household?

- Less than 1 year
- 1 year
- 2 years
- 3 years
- 4 years

You indicated that you are the primary caretaker for at least one child under age 5 that lives in your household. What is your relationship to that/those child(ren)? **Choose all that apply.**

- Parent
- Stepparent
- Grandparent
- Foster parent
- Sibling
- Aunt/Uncle
- Other

Are you now married, widowed, divorced, separated or never married?

- Married - Spouse PRESENT
- Married - Spouse ABSENT
- Widowed
- Divorced
- Separated
- Never married

Which of the following best describes your gender identity? **Choose all that apply.**

- ☐ Male
- ☐ Female
- ☐ Transgender
- ☐ Non-binary
- ☐ ______ Prefer to self-describe

What is your zipcode?

______

In what year were you born?

______

What is the highest level of formal education that you have completed? Please choose only one of the following:

- ○ Some high school (no diploma)
- ○ High school graduate (incl. GED)
- ○ Some college (no degree)
- ○ Associates degree/technical school/apprenticeship
- ○ Bachelor’s degree
- ○ Postgraduate (e.g. Master’s, PhD) / professional degree (e.g. JD)
